# Detection and genetic analysis of the enteroaggregative *Escherichia coli* heat-stable enterotoxin (EAST1) gene in clinical isolates of enteropathogenic *Escherichia coli* (EPEC) strains

**DOI:** 10.1186/1471-2180-14-135

**Published:** 2014-05-30

**Authors:** Lucas EP Silva, Tamara B Souza, Neusa P Silva, Isabel CA Scaletsky

**Affiliations:** 1 Departamento de Microbiologia, Imunologia e Parasitologia, Universidade Federal de São Paulo, Rua Botucatu, 862, 3 andar, 04023-062 São Paulo, Brazil; 2 Disciplina de Reumatologia, Universidade Federal de São Paulo, São Paulo, Brazil

**Keywords:** EAST1 gene, *astA* gene, Enteropathogenic *Escherichia coli*

## Abstract

**Background:**

The enteroaggregative *E. coli* heat-stable enterotoxin 1 (EAST1) encoded by *astA* gene has been found in enteropathogenic *E. coli* (EPEC) strains. However, it is not sufficient to simply probe strains with an *astA* gene probe due to the existence of *astA* mutants (type 1 and type 2 SHEAST) and EAST1 variants (EAST1 v1-4). In this study, 222 EPEC (70 typical and 152 atypical) isolates were tested for the presence of the *astA* gene sequence by PCR and sequencing.

**Results:**

The *astA* gene was amplified from 54 strains, 11 typical and 43 atypical. Sequence analysis of the PCR products showed that 25 strains, 7 typical and 18 atypical, had an intact *astA* gene. A subgroup of 7 atypical strains had a variant type of the *astA* gene sequence, with four non-synonymous nucleotide substitutions. The remaining 22 strains had mutated *astA* gene with nucleotide deletions or substitutions in the first 8 codons. The RT-PCR results showed that the *astA* gene was transcribed only by the strains carrying either the intact or the variant type of the *astA* gene sequence. Southern blot analysis indicated that *astA* is located in EAF plasmid in typical strains, and in plasmids of similar size in atypical strains. Strains carrying intact *astA* genes were more frequently found in diarrheic children than in non-diarrheic children (p < 0.05).

**Conclusion:**

In conclusion, our data suggest that the presence of an intact *astA* gene may represent an additional virulence determinant in both EPEC groups.

## Background

Enteropathogenic *Escherichia coli* (EPEC) are an important cause of infant diarrhea in developing countries [1]. The majority of EPEC isolates belong to classic serotypes derived from 12 classical O serogroups (O26, O55, O86, O111, O114, O119, O125, O126, O127, O128, O142, and O158) [2,3]. EPEC induces attaching and effacing (A/E) lesions on epithelial cells, characterized by microvilli destruction, cytoskeleton rearrangement, and the formation of a pedestal-like structure at the site of bacterial contact [4]. The A/E genes are localized to the locus for enterocyte effacement (LEE) and encode intimin*,* a type III secretion system, secreted proteins and the translocated intimin receptor [5-7].

“Typical” EPEC strains (tEPEC) contain also the EPEC adherence factor (EAF) plasmid [8], which carries genes encoding a regulator (*per*) [9] and the bundle-forming pili (BFP) [10]. EPEC strains lacking the EAF plasmid have been designated “atypical” EPEC (aEPEC) [11]. Recent epidemiological studies indicate that aEPEC are more prevalent than tEPEC in both developed and developing countries [1]. Some aEPEC strains are genetically related to the enterohemorrhagic *E. coli* (EHEC), and both are considered as emerging pathogens [12].

Typical EPEC strains express only the virulence factors encoded by the LEE region and the EAF plasmid, with the exception of the cytolethal distending toxin produced by O86:H34 strains and the enteroaggregative heat-stable enterotoxin 1 (EAST1) found in O55:H6 and O127:H6 strains. In contrast, aEPEC strains frequently express EAST1 and additional virulence factors not encoded by LEE region [12]. In a previous study [13], EAST1 was the most frequent (24%) virulence factor found in a collection of 65 aEPEC strains, and was significantly associated with children diarrhea.

EAST1-positive aEPEC strains have been associated with outbreaks of diarrhea involving children and adults in the United State [14] and Japan [15]. However, it is not sufficient to simply probe strains with an *astA* gene probe due to the existence of EAST1 variants [16]. In one study, 100% of the O26, O111, O145, and O157:H7 enterohemorrhagic *E. coli* (EHEC) strains examined carried DNA sequences homologous to the EAST1 gene (SHEAST) with two different mutation types. Type 1 SHEAST has 12 nucleotide non-synonymous substitutions including one in the initiation codon; type 2 SHEAST lacks the first 8 codons of EAST1 sequence [16]. The focus of the study was to investigate the *astA* gene sequence present in tEPEC and aEPEC strains. The strains were collected in different cities of Brazil in different periods of time and in a previous study poor relatedness was observed by RAPD analysis of 118 strains belonging to this collection [13].

## Results and discussion

We examined 222 EPEC strains (70 typical and 152 atypical) for the presence of the *astA* gene by PCR using primers that anneal to the 5’ ends of the EAEC 042 *astA* gene sequence [16]. Those strains were isolated from diarrheic and non diarrheic Brazilian children in previous studies [17-20]. As shown in Table 1, 11 (16%) tEPEC and 43 (28%) aEPEC strains were positive in the PCR assay. Among the aEPEC PCR-positive strains, 13 belonged to the O26 and O119 serogroups.

**Table 1:** **EPEC-*astA *** **strains isolated from diarrheic and non-diarrheic children**

**EPEC**	**Serotype**	**No. of strains (positive**/**total)**		
		**Diarrheic children**	**Non-diarrheic children**	**Total of children**
**tEPEC**	O55:NM;HND	0/13	0/1	0/14
	O86:NM;H34	0/2	0	0/2
	O111:NM;H2,HND	4/9	0	4/9
	O119:NM;H6;HND	2/22	0/3	2/25
	O127:NM;H6	0/1	2/3	2/4
	Other serotypes^a^	3/14	0/2	3/16
Subtotal		9/61	2/9	11/70
**aEPEC**	O26:H11;HND	6/10	0/2	6/12
	O55:HND	2/3	1/2	3/5
	O111:NM	2/2	1/2	3/4
	O114:NM	0	0/1	0/1
	O119:H2;HND	7/9	0/3	7/12
	O126:NM	0/1	0	0/1
	O127:NM;H40	0/3	0/1	0/4
	O128:NM	0/3	0	0/3
	O142:NM;H2	1/8	0	1/8
	Other serotypes^b^	18/68	5/34	23/102
Subtotal		36/107	7/45	43/152
**Total**		**45/168**	**9/54**	**54/222**

^a^O2:H2;H45; O101:H33; O145:HND; O157:HND; O162:H33; ONT:H45; ONT:HND.

^b^O4:HND; O15:HND O33:H6; O35:H19; O37:HND; O49:HND; O61:HND; O63:HND; O79:HND; O85:H40; O96:HND; O98:HND; O101:NM; O103:NM; O105:H7; O108:H31; O109:H54; O117:HND; O132:HND; O141:HND; O1523H2; O156:H16; O157:HND; O167:H6; O169:H6; O175:HND;ONT:NM; ONT:H18; ONT:HND.

Note: NM, nommotile, ND, nondetermined, ONT, nontypeable.

**Figure 1:**
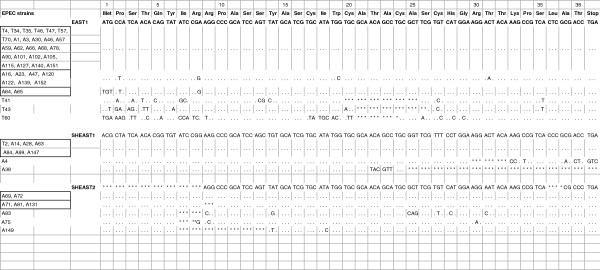
**Nucleotide sequences of the PCR products from tEPEC (T) and aEPEC (A) strains.** The nucleotide sequences of the EAST1, SHEAST1 and SHEAST2 genes are shown for comparison. Identical nucleotides are shown as dots. Asterisks indicate the positions of nucleotide deletion.

The 54 *astA* gene PCR products were sequenced. Twenty five strains, 7 tEPEC and 18 aEPEC, carried the DNA sequence identical to the EAST1 gene (042-type EAST1) (Figure 1). A subgroup of 7 aEPEC strains presented a variant type of the 042-type EAST1 gene sequence, with four non-synonymous nucleotide substitutions. Nine other strains, including one typical, carried either the sequence identical to type 1 SHEAST (7 strains) or to type 2 SHEAST (two strains). The remaining 13 strains carried mutated sequences of the 042-type EAST1 (five strains)*,* type 1 SHEAST (two strains) or type 2 SHEAST (six strains) genes.

**Figure 2:**
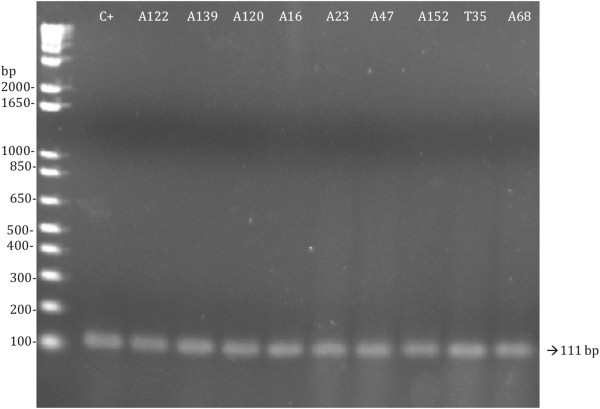
**Agarose gel electrophoresis of the RT-PCR products of representative strains of tEPEC (T) and aEPEC (A).** EAEC 042 strain (C+) was used as positive control. Molecular size standard bands are at left.

The expression of EAST1 was examined by RT-PCR and quantitative RT-PCR. The RT-PCR results showed that the *astA* gene was transcribed only by the strains carrying either the intact or the variant type of the *astA* gene sequence (Figure 2). The *astA* gene expression levels of the 32 RT-PCR positive strains (CT values ranged from 20.3 ± 0.11 to 21.6 ± 0.04) were nearly identical to that of EAEC 042 strain (CT value 20.8 ± 0.01).

**Figure 3:**
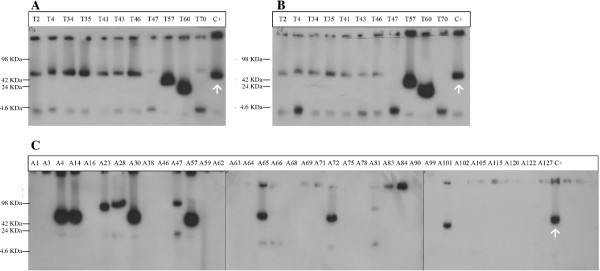
**Southern blot hybridization of the plasmids of tEPEC (T) and aEPEC (A) strains. (A)** and **(C)** Hybridization results with the *astA* probe. **(B)** Hybridization results with the EAF probe. EAEC 042 and EPEC E2348/69 were used as positive controls (C+) for *astA* and EAF probes, respectively. The arrows in panels **A** and **C** indicate the pAA2 plasmid (65-MDa) for EAEC 042 strain and the arrow in panel **B** indicate the EAF plasmid (60-MDa) for EPEC E2348/69 strain. Molecular size standard bands are at left.

Plasmids of the 54 PCR-positive strains were examined for *astA* gene presence by Southern blot hybridization with the *astA* probe. In 23 (42.6%) strains, a single copy of the *astA* gene was located to a large plasmid (Figure 3). In all the eleven tEPEC strains, the *astA* probe hybridized to the EAF plasmid as previously reported [21], and in twelve aEPEC the *astA* probe hybridized with large plasmids of similar size. The plasmids of the remaining strains were *astA* probe negative.

We previously reported that 24% of 65 aEPEC strains hybridized with a DNA probe for EAST1 [13]. Here, we analyzed by PCR a larger group of EPEC, including typical strains and found that 11 (16%) of 70 tEPEC and 43 (28%) of 152 aEPEC were *astA* positive. Sequence analysis of the PCR products showed that 7 (63.6%) of 11 tEPEC and 18 (41.9%) of 43 aEPEC had an intact 042-type *astA* gene.

As shown in Table 2, strains carrying intact *astA* gene were more frequently found in diarrheic children than in non-diarrheic children (p = 0.03, Fisher’s exact test). However, we should point out that among the 222 strains analyzed only 118 were collected from a case–control study [13].

**Table 2:** **Sequences of the *astA *** **gene found in EPEC strains isolated from diarrheic and non-diarrheic children**

***astA *** **gene sequence type**	**N (%) of strains from:**		**Serogroup ( *n *)**
	**Diarrheic children**	**Non-diarrheic children**	
042-type EAST1	24 (14.3)	1 (1.8)^a^	O9 (1), O33 (2), O108 (2), O111 (1), O119 (8), O142 (1), O152 (1), O157 (1), O169 (1), OND (7)
EAST1v5	6 (3.6)	1 (1.8)	O26 (1), O9 (1), O96 (1) O111 (1), O141 (1), ONT (2)
type 1 SHEAST	6 (3.6)	1 (1.8)	O26 (1), O55 (1), O103 (1), O153 (1), OND (3)
type 2 SHEAST	2 (1.2)	0	O26 (1), O55 (1)
mutant	7	6	O26 (3), O55 (1), O111 (5), O119 (1), O127 (2), ONT (1)
Total	45	9	

^a^p = 0.03; Fisher’s exact test (diarrheic x non-diarrheic).

**Figure 4:**
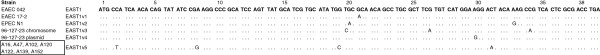
**Nucleotide sequence of the EAST1 gene and its variants, including the new one described in this study.** Identical nucleotides are shown as dots.

The EAST1 gene family includes one major type of sequence, i.e. the *astA* of EAEC strain 042 that is widely distributed among different diarrheagenic *E. coli* strains [21-26] and four variant types of EAST1, i.e. the EAST1v1 of EAEC 17–2 [21,22], EAST1v2 of EPEC N1 [21], and EAST1v3 and EAST1v4 of *E. coli* O166:H15 [25].In this study, a subgroup of aEPEC strains had a new variant type of EAST1 gene sequence that differed from those previously reported, and was denominated EAST1v5 (Figure 4). The RT-PCR analysis showed that EAST1v5 was transcribed to produce mRNA. However, more studies are necessary to determine whether EAST1v5 is associated with a functional polypeptide toxin.

## Conclusion

In conclusion, our data suggest that the presence of an intact *astA* gene may represent an additional virulence determinant in both EPEC groups.

## Methods

### Bacterial strains

The 222 EPEC strains examined in this study included 176 strains isolated in 1999 to 2004 during an epidemiological study of acute diarrhea in children <2 years of age conducted in different regions of Brazil, and 46 strains isolated from children <5 years of age with diarrhea in São Paulo between 2002 to 2003 [17-20]. All strains were characterized as tEPEC or aEPEC by hybridization with *eae* and EAF probes and serotyped (Table 1).

### Ethics statement

The study was approved by the ethics committee of the Universidade Federal de São Paulo, Brazil. Stool samples were obtained with the written informed consent from the parents or guardians of the children.

### PCR assays

For template DNA preparation, three to five isolated bacterial colonies grown on LB agar plates were pooled, suspended in 300 μl of sterile distilled water, and boiled for 10 min. PCR was carried out in a total volume of 25-μl containing 5 μl of template DNA. PCR primers were EAST13a (F-5’AGAACTGCTGGGTATGTGGCT) located 110 nucleotides upstream from the initiation ATG sequence of the *astA* gene, and EAST12b (R-5’CTGCTGGCCTGCCTCTTCCGT) located 20 nucleotides downstream from the stop TGA sequence of the *astA* gene [26]. Cycling conditions were denaturation for 30 s at 95°C, annealing for 120 s at 55°C, and polymerization for 120 s at 72°C (30 cycles). PCR products were analyzed by 2% agarose gel electrophoresis.

### DNA hybridization

The following probes were used in this study: *astA*, a 111-bp PCR product from EAEC 042 strain with the primer set EAST11a (5’-CCATCAACACAGTATTCCGA) and EAST12b (5’-GGTCGCGAGTGACGGCTTTGT) [26]; and EAF, a 1.0 kb *BamH*I-*Sal*I fragment from plasmid pMAR2 [27]. The DNA fragments were purified, labeled with [α-^32^P] dCTP with a DNA labeling kit (Amersham Pharmacia Biotech Inc., EUA) and used as probes. For Southern blotting, plasmid DNA was extracted using the method of Birnboim and Doly [28], separated in 0.8% agarose gel electrophoresis, and transferred to a nylon membrane, following a standard protocol [29]. Blots were hybridized in a solution containing the labeled probe (10^5^ cpm), 5 × standard saline citrate (SSC), 2 × Denhardt’s solution (Invitrogen), 0.1% sodium dodecyl sulfate (SDS), and 5 mg/ml of salmon sperm DNA for 16 h at 65°C. After hybridization, washes were done in aqueous solution with 2 × SSC with 0.1% SDS and exposed to X-ray film.

### RNA extraction and RT-PCR assays

Total RNA was extracted after bacterial growth in LB broth for 18 h at 37°C with the RNase Mini extraction kit (Qiagen) according to the manufacturer’s instructions. After extraction, approximately 1 μg of total RNA was digested with DNase I (Qiagen) for 30 min at 37°C, and the enzyme was then inactivated by adding 1 μl of 25 mM EDTA and heating the solution at 65°C for 10 min. To obtain the cDNA, the SperScript III One Step RT-PCR System with Platinum *Taq* DNA polymerase (Invitrogen) was used according to the manufacturer’s specifications. Primers for 16S ribosomal protein were used to control PCR [30], and the assay was then carried out with the primers EAST11a and EAST11b [26]. PCR products were analyzed by 2% agarose gel electrophoresis.

Quantitative PCR was performed in a Mastercycler ep realplex^4^ (Eppendorf), and threshold cycle numbers were determined using Eppendorf realplex software (version 2.0). Reactions were performed in triplicate, and threshold cycle numbers were averaged. The 50-μl reaction mixture was prepared as follows: 25 μl of Platinum® Quantitative PCR SuperMix-UDG (Invitrogen), 10 μM of the Taqman probe (5’FAM-TGCATCGTGCATATGGTGCGCAA) and 10 μM of each primer (R-5’GCGAGTGACGGCTTTGTAG and F-5’GAAGGCCCGCATCCAGTT), and 10 μl of cDNA (100 ng). The reaction consisted of: 2 min at 48°C; 10 min at 95°C followed by 40 cycles of 15 s at 95°C, 1 min at 60°C, and 1 min at 72°C. The *astA* expression of the tested strains was compared to the *astA* expression of EAEC 042, according to the formula, 2^(-ΔΔCt)^[31].

### DNA sequencing

Nucleotide sequencing of the PCR products was performed at the Centro de Estudos do Genoma Humano-USP, São Paulo. Nucleotide sequence data were analyzed using SeqMan and MegAlign software and the BLAST tool (http://www.ncbi.nlm.nih.gov/BLAST).

### Statistical analysis

Data for diarrheic and non diarrheic children were compared using a 2-tailed Chi-square test. Results with p values ≤ 0.05 were considered to be statistically significant.

### Nucleotide sequence and accession number

The EAST1v5 gene sequence was deposited in the NCBI database under accession number KJ47188.

## Competing interests

The authors declare that they have no competing interests.

## Authors’ contributions

LEPS and TBS performed experiments and analyzed data. NPS and ICAS wrote the manuscript. All authors read and approved the final manuscript.

## References

[B1] Ochoa TJ Contreras CA Enteropathogenic *Escherichia coli* infection in children Curr Opin Infect Dis 2011 24 478 483 10.1097/QCO.0b013e32834a8b8b 21857511 PMC3277943

[B2] WHO Programme for Control of Diarrhoeal Diseases, Manual for Laboratory Investigation of Acute Enteric Infections 1987 Geneva: World Health Organization

[B3] Nataro JP Kaper JB Diarrheagenic *Escherichia coli* Clin Microbiol Rev 1998 11 142 201 9457432 10.1128/cmr.11.1.142 PMC121379

[B4] Moon HW Whipp SC Argenzio RA Levine MM Gianella RA Attaching and effacing activities of rabbit and human enteropathogenic *Escherichia coli* in pig and rabbit intestines Infect Immun 1983 41 1340 1351 6350186 10.1128/iai.41.3.1340-1351.1983 PMC264644

[B5] Jerse AE Yu J Tall BD Kaper JB A genetic locus of enteropathogenic *Escherichia coli* necessary for the production of attaching and effacing lesions on tissue culture cells Proc Natl Acad Sci U S A 1990 87 7839 7843 10.1073/pnas.87.20.7839 2172966 PMC54845

[B6] Jarvis KG Girón JA Jerse AE McDaniel TK Donnenberg MS Kaper JB Enteropathogenic *Escherichia coli* contains a putative type III secretion system necessary for the export of proteins involved in attaching and effacing lesion formation Proc Natl Acad Sci U S A 1995 92 7996 8000 10.1073/pnas.92.17.7996 7644527 PMC41273

[B7] Kenny B DeVinney R Stein M Finlay BB Enteropathogenic *E. coli* (EPEC) transfers its receptor for intimate adherence into mammalian cells Cell 1997 91 511 520 10.1016/S0092-8674(00)80437-7 9390560

[B8] Baldini MM Kaper JB Levine MM Candy DC Moon HW Plasmid-mediated adhesion in enteropathogenic *Escherichia coli* J Pediatr Gastroenterol Nutr 1983 2 534 539 10.1097/00005176-198302030-00023 6352891

[B9] Gómez-Duarte OG Kaper JB A plasmid-encoded regulatory region activates chromosome *eae*A expression in enteropathogenic *Escherichia coli* Infect Immun 1995 63 1767 1776 7729884 10.1128/iai.63.5.1767-1776.1995 PMC173222

[B10] Girón JA Ho AS Schoolnik GK An inducible bundle-forming pilus of enteropathogenic *Escherichia coli* Science 1991 254 710 713 10.1126/science.1683004 1683004

[B11] Kaper JB Defining EPEC Rev Microbiol São Paulo 1996 27 130 133

[B12] Trabulsi LR Keller R Gomes TAT Typical and atypical Enteropathogenic *Eschericia coli* (EPEC) Emerg Infect Dis 2002 8 508 513 10.3201/eid0805.010385 11996687 PMC2732489

[B13] Dulguer MV Fabricotti SH Bando SY Moreira-Filho CA Fagundes-Neto U Scaletsky ICA Atypical enteropathogenic *Escherichia coli* strains: phenotypic and genetic profiling reveals a strong association between enteroaggregative *E. coli* heat-stable enterotoxin and diarrhea J Infect Dis 2003 188 1685 1694 10.1086/379666 14639540

[B14] Hedberg CW Savarino SJ Besser JB Paulus CJ Thelen VM Myers LJ Cameron DN Barret TJ Kaper JB Osterholm MT An outbreak of foodborne illness caused by *Escherichia coli* O39:NM, an agent not fitting into the existing scheme for classifying diarrheogenic *E. coli* J Infect Dis 1997 176 1625 1628 10.1086/517342 9395379

[B15] Yatsuyanagi Y Salto S Miyajima T Characterization of atypical enteropathogenic *Escherichia coli* strains harboring the *astA* gene that were associated with a waterborne outbreak of diarrhea in Japan J Clin Microbiol 2003 41 2033 2039 10.1128/JCM.41.5.2033-2039.2003 12734245 PMC154716

[B16] Yamamoto T Taneike I The sequences of enterohemorrhagic *Escherichia coli* and *Yersinia pestis* that are homologous to the enteroaggregative *E. coli* heat-stable enterotoxin gene: cross-species transfer in evolution FEBS Lett 2000 472 22 26 10.1016/S0014-5793(00)01414-9 10781798

[B17] Scaletsky ICA Fabbricotti SH Aranda KR Morais MB Fagundes-Neto U Comparison of DNA hybridization and PCR assays for detection of putative pathogenic enteroadherent *Escherichia coli* J Clin Microbiol 2002 40 1254 1258 10.1128/JCM.40.4.1254-1258.2002 11923341 PMC140355

[B18] Scaletsky ICA Fabbricotti SH Silva SO Morais MB Fagundes-Neto U HEp-2–adherent *Escherichia coli* strains associated with acute infantile diarrhea, São Paulo, Brazil Emerg Infect Dis 2002 8 855 858 10.3201/eid0808.010492 12141974 PMC2732515

[B19] Araújo JM Tabarelli GF Aranda KR Fabbricotti SH Fagundes-Neto U Mendes CM Scaletsky ICA Typical enteroaggregative and atypical enteropathogenic types of *Escherichia coli* (EPEC) are the most prevalent diarrhea-associated pathotypes among Brazilian children J Clin Microbiol 2007 45 3396 3399 10.1128/JCM.00084-07 17670930 PMC2045331

[B20] Scaletsky ICA Aranda KR Souza TB Silva NP Morais MB Evidence of pathogenic subgroups among atypical enteropathogenic *Escherichia coli* strains J Clin Microbiol 2009 47 3756 3759 10.1128/JCM.01599-09 19759223 PMC2772600

[B21] Yamamoto T Wakisaka N Sato F Kato A Comparison of the nucleotide sequence of enteroaggregative *Escherichia coli* heat-stable enterotoxin 1 genes among diarrhea-associated *Escherichia coli* FEMS Microbiol Lett 1997 147 89 96 10.1111/j.1574-6968.1997.tb10225.x 9037769

[B22] Savarino SJ McVeigh A Watson J Cravioto A Molina J Echeverria P Bhan MK Levine MM Fasano A Enteroaggregative *Escherichia coli* heat-stable enterotoxin is not restricted to enteroaggregative *E. coli* J Infect Dis 1996 173 1019 1022 10.1093/infdis/173.4.1019 8603943

[B23] Sousa CP Dubreuil JD Distribution and expression of the *astA* gene (EAST1 toxin) in *Escherichia coli* and *Salmonella* Int J Med Microbiol 2001 291 15 20 10.1078/1438-4221-00097 11403406

[B24] Savarino SJ Fasano A Watson J Martin BM Levine MM Guandalini S Guerry P Enteroaggregative *Escherichia coli* heat-stable enterotoxin 1 represents another subfamily of *E. coli* heat-stable toxin Proc Natl Acad Sci U S A 1993 90 3093 3097 10.1073/pnas.90.7.3093 8385356 PMC46243

[B25] Zhou Z Ogasawara J Nishikawa Y Seto Y Helander A Hase A Iritani N Nakamura H Arikawa K Kai A Kamata Y Hoshi H Haruki K An outbreak of gastroenteritis in Osaka, Japan due to *Escherichia coli* serogroup O166:H15 that had a coding gene for enteroaggregative *E. coli* heat-stable enterotoxin 1 (EAST1) Epidemiol Infect 2001 128 363 371 12113479 10.1017/s0950268802006994 PMC2869831

[B26] Yamamoto T Echeverria P Detection of the enteroaggregative *Escherichia coli* heat- stable enterotoxin 1 gene sequences in enterotoxigenic *E. coli* strains pathogenic for humans Infect Immun 1996 64 1441 1445 8606115 10.1128/iai.64.4.1441-1445.1996 PMC173940

[B27] Nataro JP Baldini MM Kaper JB Black RE Bravo N Levine MM Detection of an adherence factor of enteropathogenic *Escherichia coli* with a DNA probe J Infect Dis 1985 152 560 565 10.1093/infdis/152.3.560 2863319

[B28] Birnboim HC Doly J A rapid alkaline extraction procedure for screening recombinant plasmid DNA Nucleic Acids Res 1979 247 1513 1523 388356 10.1093/nar/7.6.1513 PMC342324

[B29] Sambrook J Fritsch EF Maniatis T Molecular Cloning: A Laboratory Manual 1989 2 Cold Spring Harbor, NY: Cold Spring Harbor Laboratory Press

[B30] Leverton LQ Kaper JB Temporal expression of enteropathogenic *Escherichia coli* virulence genes in an in vitro model of infection Infect Immun 2005 73 1034 1043 10.1128/IAI.73.2.1034-1043.2005 15664947 PMC546935

[B31] Livak KJ Schmittgen TD Analysis of relative gene expression data using real-time quantitative PCR and the 2(-Delta Delta C(T)) method Methods 2001 25 402 408 10.1006/meth.2001.1262 11846609

